# Supplementary material for: Moderate confirmation bias enhances decision-making in groups of reinforcement-learning agents
Source: PLoS Comput Biol. 2024 Sep 4;20(9):e1012404. doi: 10.1371/journal.pcbi.1012404 (PMC11404843; doi:10.1371/journal.pcbi.1012404)
Supplement: S1 Text — (PDF) [file pcbi.1012404.s001.pdf]

S1 Text. Effect of higher temperature (lower β)

To get an overview of our model's behavior when agents are more exploratory, we ran additional simulations with lower values of β. First of all, we used CARL's deterministic model (define in Section IV A 4) to get an overview of how β impacts polarization after convergence in a group of two agents. To this end, we computed the difference between both agents' Q-value gaps after 1000 trials. The higher this difference, the higher the polarization. We varied β from 0 to 20, and found an interesting landscape of transitions between polarization regimes in all environments. As can be seen in S6 Fig, agents exhibit consensus for low values of β in all environments, but end up getting polarized in all environments after a critical β. For $\beta = 4$ — i.e., the inverse temperature we used throughout our study —, polarization is much lower in poor environments than in mixed and rich ones. But, after $\beta = 5$, agents become significantly more polarized in a poor environment.

From there, it is natural to ask how agents perform when β is so low as to prevent polarization. Thus, we ran additional simulations with lower values of β, in the same conditions as in Fig 1 (although we varied group size from 1 to 10 instead of 1 to 20, as it did not seem necessary to go further). We investigated $\beta = 1$ and $\beta = 0.5$. As seen in S6 Fig, these inverse temperatures should not give rise to polarization in any environment. We predicted that, because these lower values of β reintroduce exploration into the system, agents would more easily get unstuck from suboptimal performance regimes, which, in turn, would cause small groups of confirmatory agents to perform significantly better in rich environments. Results are shown in S7 Fig.

A look at final Q-value gap distributions (S7G-I Fig) suggests that, for these lower values of β, polarization indeed disappears in all environments. However, as shown in S7C Fig, small groups of agents with a confirmation bias still appear to perform less well than unbiased agents in rich environments, although this effect becomes barely visible when $\beta = 0.5$ (S7F Fig). A more thorough analysis with the deterministic model confirms that lower inverse temperature is not beneficial to small groups of agents in rich environments (S8 Fig). In fact, despite the absence of polarization, confirmatory agents in rich environments end up with a lower Q-value gap than the difference between both options' expected values. Observing agents' final Q-values in a poor, and in a rich, environment (S9 Fig), one notices the following:

- In a rich environment, the best option's Q-value is slightly inflated compared to its

expected value; whereas the worst option's Q-value is very inflated.

- In a poor environment, the best option's Q-value is very inflated compared to its expected value; whereas the worst option's Q-value is only slightly inflated.

In their simulation study, Lefebvre et al. (2022) showed that, for individual agents, a confirmation bias is beneficial because it allows an individual to overestimate the best option's Q-value, while underestimating the worst option's Q-value [1]. The aforementioned results suggest that, for our agents, this is not the case: by inflating both options' Q-values, they behave more similarly to optimistic agents. This is reminiscent of another simulation study, by Cazé and Van der Meer (2013). In this work, the authors studied the impact of optimism and pessimism on single agents' performance in a two-armed bandit task. They demonstrated that optimism is beneficial in poor environments, while pessimism is beneficial in rich environments. The reason for such an effect is mathematical: since Q-values are bounded between -1 and 1, an optimistic estimate of the expected value saturates as it approaches 1, and a pessimistic estimate saturates as it approaches -1. As a consequence, optimistic agents end up with a smaller Q-value gap in the rich domain, but a wider gap in the poor domain; whereas pessimistic agents exhibit the opposite pattern [2].

The question remains why our two confirmatory agents behave like optimistic agents. The reason is that, when it comes to confirmation bias, exploration is not enough: *who* explores matters. Indeed, a confirmatory agent tends to inflate the Q-value of the option it chooses, and deflate the Q-value of the option it does not choose – in short, a confirmatory agent is optimistic about chosen options, and pessimistic about unchosen options. When inverse temperature decreases, an agent tends to sample alternative options more. Subsequently, it has more opportunities to be optimistic about both options, thus inflating both Q-values. On the other hand, the opportunities to deflate the other option's Q-value are rare: this happens when the second agent chooses the latter option and receives an outcome of -1, which, in a rich environment, does not happen often. Therefore, the lower the inverse temperature, the more an agent becomes optimistic about the worst option; this results in a narrower final Q-value gap.

When one increases group size (S9 Fig), one observes that the lowest Q-value is not inflated anymore. Indeed, with a sufficiently large group in a rich environment, an agent has more opportunities to deflate the worst option's Q-value – it is more likely that any other

agent will choose that option and get an outcome of -1.

To sum up, the drop in performance in small groups of confirmatory agents in rich environments is due to two different effects:

- in a consensus regime (low β): agents do not have enough opportunities to get negative counterfactual feedback and, thus, inflate the worst option's Q-value. They behave optimistically;

- in a polarization regime (high β): on top of the aforementioned, "optimism" effect, agents get polarized, with some of them performing very well and some others, very poorly. On average, they perform worse than an equivalent group of unbiased agents.

The effect we underlined is reminiscent of Denrell & March's "hot stove effect" (2001). This effect occurs when, because of negative outcomes during the first trials, agents undervalue an option and end up neglecting it, although sampling it would correct this undervaluation [3]. As we have shown before, the drop in performance for small groups of confirmatory agents in a rich environment does not stem from reduced sampling *per se*, but from reduced *counterfactual* sampling. This reduced counterfactual sampling results in optimistic updating of both Q-values, which shrinks Q-value gaps in highly rewarding environments.

REFERENCES

1. Lefebvre, G., Summerfield, C., & Bogacz, R. (2022). A normative account of confirmation bias during reinforcement learning. Neural computation, 34(2), 307-337.

2. Cazé, R. D., & van der Meer, M. A. (2013). Adaptive properties of differential learning rates for positive and negative outcomes. Biological cybernetics, 107(6), 711-719.

3. Denrell, J., & March, J. G. (2001). Adaptation as information restriction: The hot stove effect. Organization science, 12(5), 523-538.
