# Supplementary material for: Moderate confirmation bias enhances decision-making in groups of reinforcement-learning agents
Source: PLoS Comput Biol. 2024 Sep 4;20(9):e1012404. doi: 10.1371/journal.pcbi.1012404 (PMC11404843; doi:10.1371/journal.pcbi.1012404)
Supplement: S1 Fig — (PDF) [file pcbi.1012404.s002.pdf]

S1 Fig. Mean collected payoff per trial for one agent in the group.

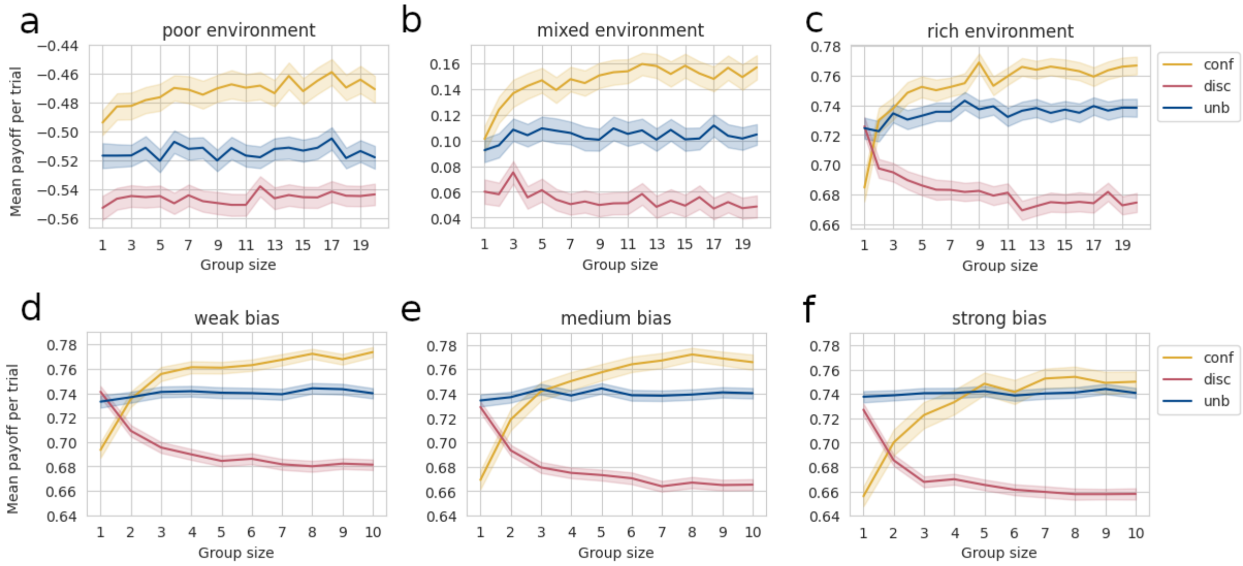

FIG. S1. A-C: Mean collected payoff per trial for one agent in the group, as a function of group size, bias type, and resource scarcity. A: poor environment: $p_1 = 0.3$, $p_2 = 0.1$. B: mixed environment: $p_1 = 0.6$, $p_2 = 0.4$. C: rich environment: $p_1 = 0.9$, $p_2 = 0.7$. Yellow curve: confirmatory agent; blue curve: unbiased agent; red curve: disconfirmatory agent. Transparent zone denotes 95%-confidence intervals. For each combination of conditions, $N_{simu} = 500$ simulations were run. Within each simulation, agents perform the task over $N_{trials} = 100$ trials — i.e., they activate the bandit 100 times. D-F: Mean collected payoff per trial for one agent in the group, as a function of group size, bias type, and bias strength in a rich environment. D: weak bias: $b = 3$ for confirmatory agents, $b = \frac{1}{3}$ for disconfirmatory agents. E: medium bias: $b = \frac{17}{3}$ for confirmatory agents, $b = \frac{3}{17}$ for disconfirmatory agents. F: strong bias: $b = 9$ for confirmatory agents, $b = \frac{1}{9}$ for disconfirmatory agents. For each combination of conditions, $N_{simu} = 500$ simulations were run. Within each simulation, agents perform the task during $N_{trials} = 200$ trials.
