# Supplementary material for: Moderate confirmation bias enhances decision-making in groups of reinforcement-learning agents
Source: PLoS Comput Biol. 2024 Sep 4;20(9):e1012404. doi: 10.1371/journal.pcbi.1012404 (PMC11404843; doi:10.1371/journal.pcbi.1012404)
Supplement: S2 Fig — (PDF) [file pcbi.1012404.s003.pdf]

S2 Fig. Distributions of final Q-value gaps for one agent across 1000 simulations, as a function of bias strength.

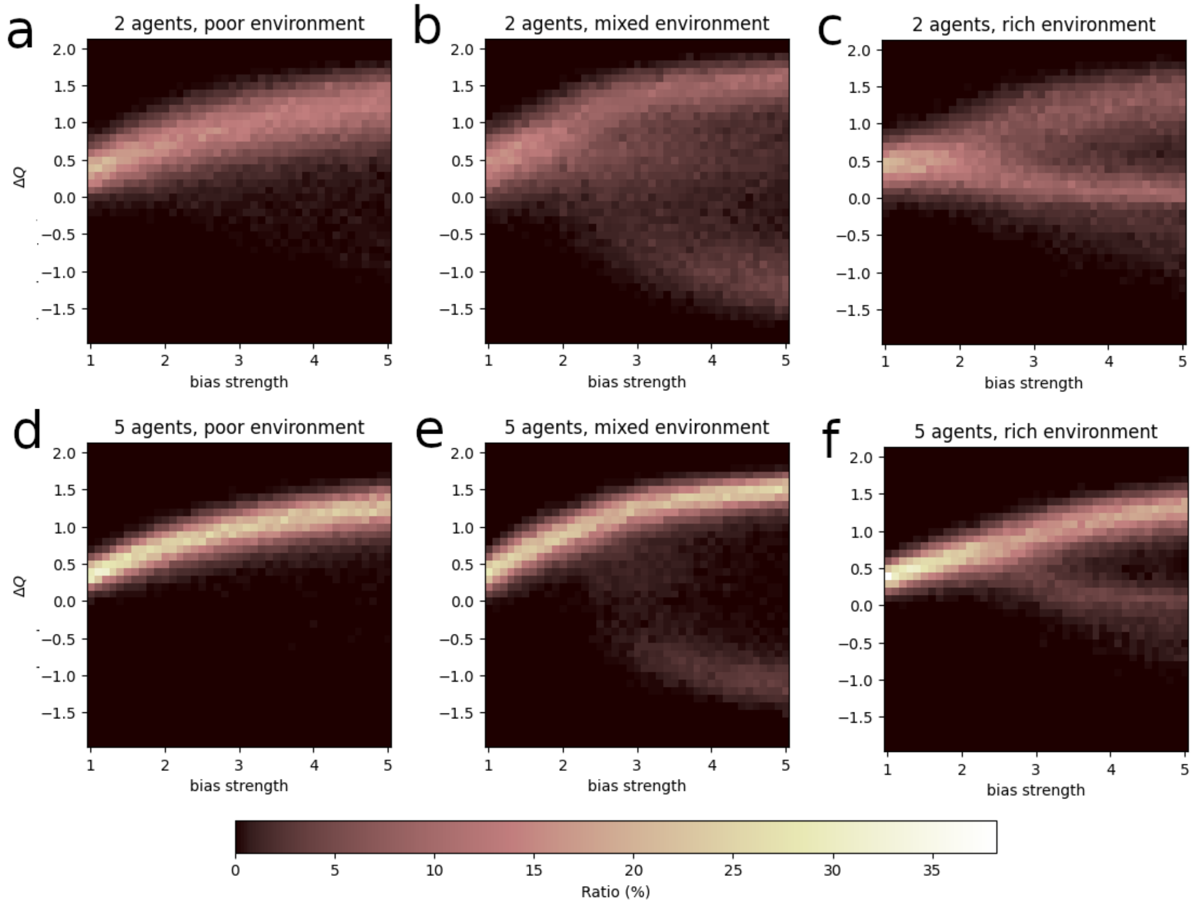

FIG. S2. Distributions of final Q-value gaps for one agent across 1000 simulations, as a function of bias strength. Color denotes ratio of simulations in which the agent ended up with a given Q-value gap. A: 2 agents, poor environment; B: 2 agents, mixed environment; C: 2 agents, rich environment; D: 5 agents, poor environment; E: 5 agents, mixed environment; F: 5 agents, rich environment.
