# Supplementary material for: Moderate confirmation bias enhances decision-making in groups of reinforcement-learning agents
Source: PLoS Comput Biol. 2024 Sep 4;20(9):e1012404. doi: 10.1371/journal.pcbi.1012404 (PMC11404843; doi:10.1371/journal.pcbi.1012404)
Supplement: S3 Fig — (PDF) [file pcbi.1012404.s004.pdf]

S3 Fig. Mean final Q-value gaps for one agent across 1000 simulations, as a function of bias strength.

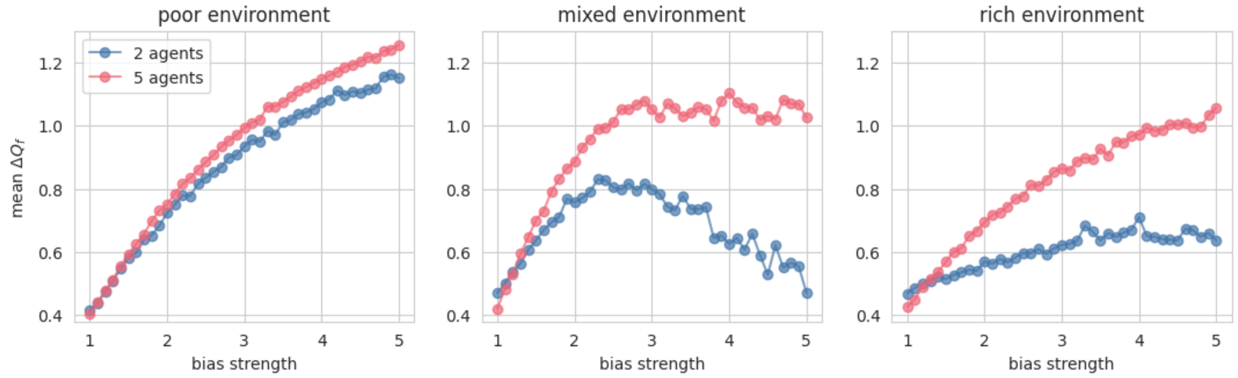

FIG. S3. Mean final Q-value gaps for one agent across 1000 simulations, as a function of bias strength. A: In a poor environment; B: in a mixed environment; C: in a rich environment.
