# Supplementary material for: Moderate confirmation bias enhances decision-making in groups of reinforcement-learning agents
Source: PLoS Comput Biol. 2024 Sep 4;20(9):e1012404. doi: 10.1371/journal.pcbi.1012404 (PMC11404843; doi:10.1371/journal.pcbi.1012404)
Supplement: S4 Fig — (PDF) [file pcbi.1012404.s005.pdf]

S4 Fig. Distributions of differences between agent 0's and other agents' Q-value gaps in a group of 5 agents, as a function of bias strength.

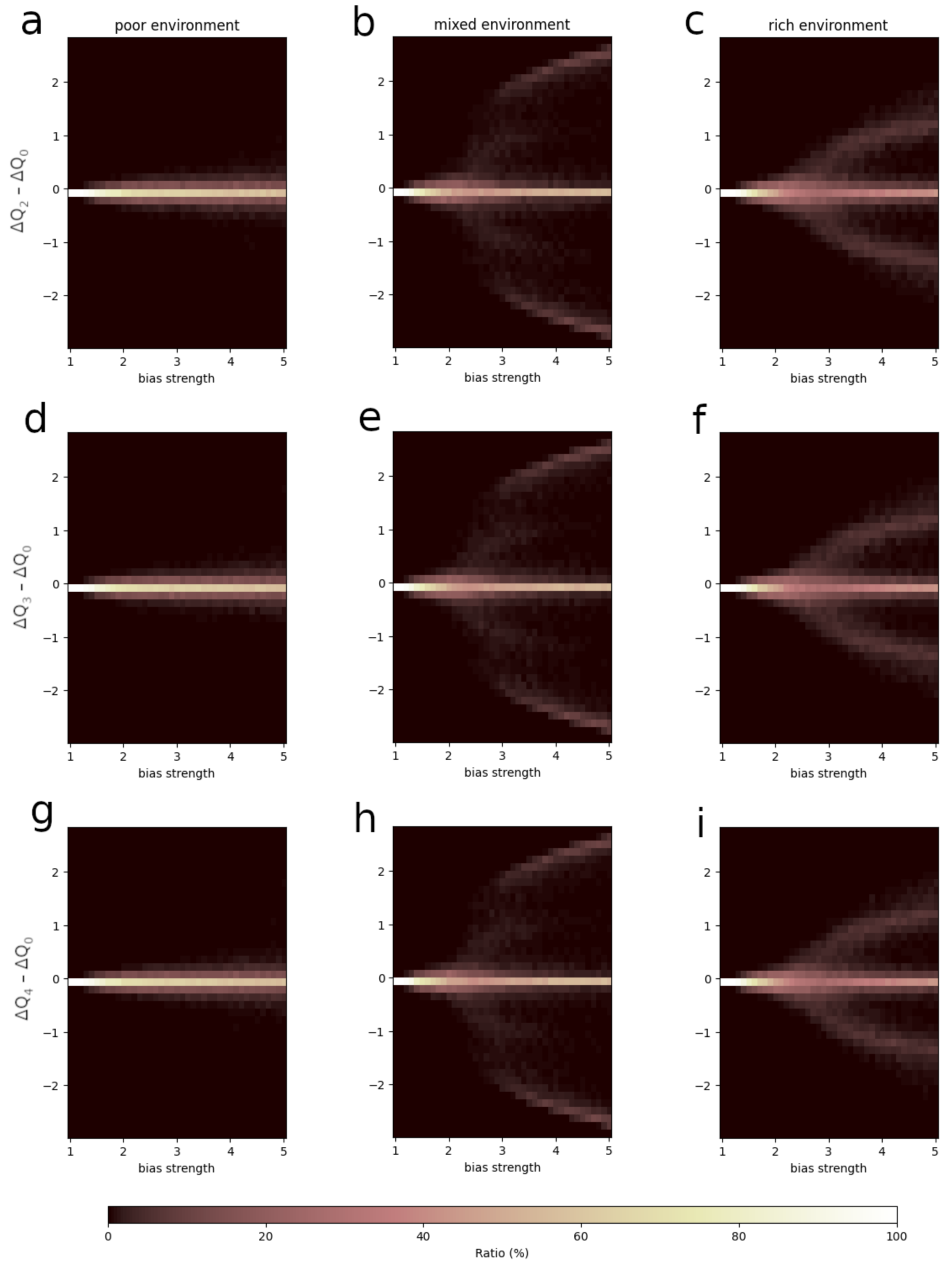

FIG. S4. Distributions of differences between agent 0's and other agents' Q-value gaps in a group of 5 agents, as a function of bias strength. A-C: agent 0 vs. agent 2 in a poor (A), mixed (B), and rich (C) environment; D-F: agent 0 vs. agent 3 in a poor (D), mixed (E), and rich (F) environment; G-I: agent 0 vs. agent 4 in a poor (G), mixed (H), and rich (I) environment.
