# Supplementary material for: Moderate confirmation bias enhances decision-making in groups of reinforcement-learning agents
Source: PLoS Comput Biol. 2024 Sep 4;20(9):e1012404. doi: 10.1371/journal.pcbi.1012404 (PMC11404843; doi:10.1371/journal.pcbi.1012404)
Supplement: S5 Fig — (PDF) [file pcbi.1012404.s006.pdf]

S5 Fig. Agents' Q-value gaps over time according to the deterministic model.

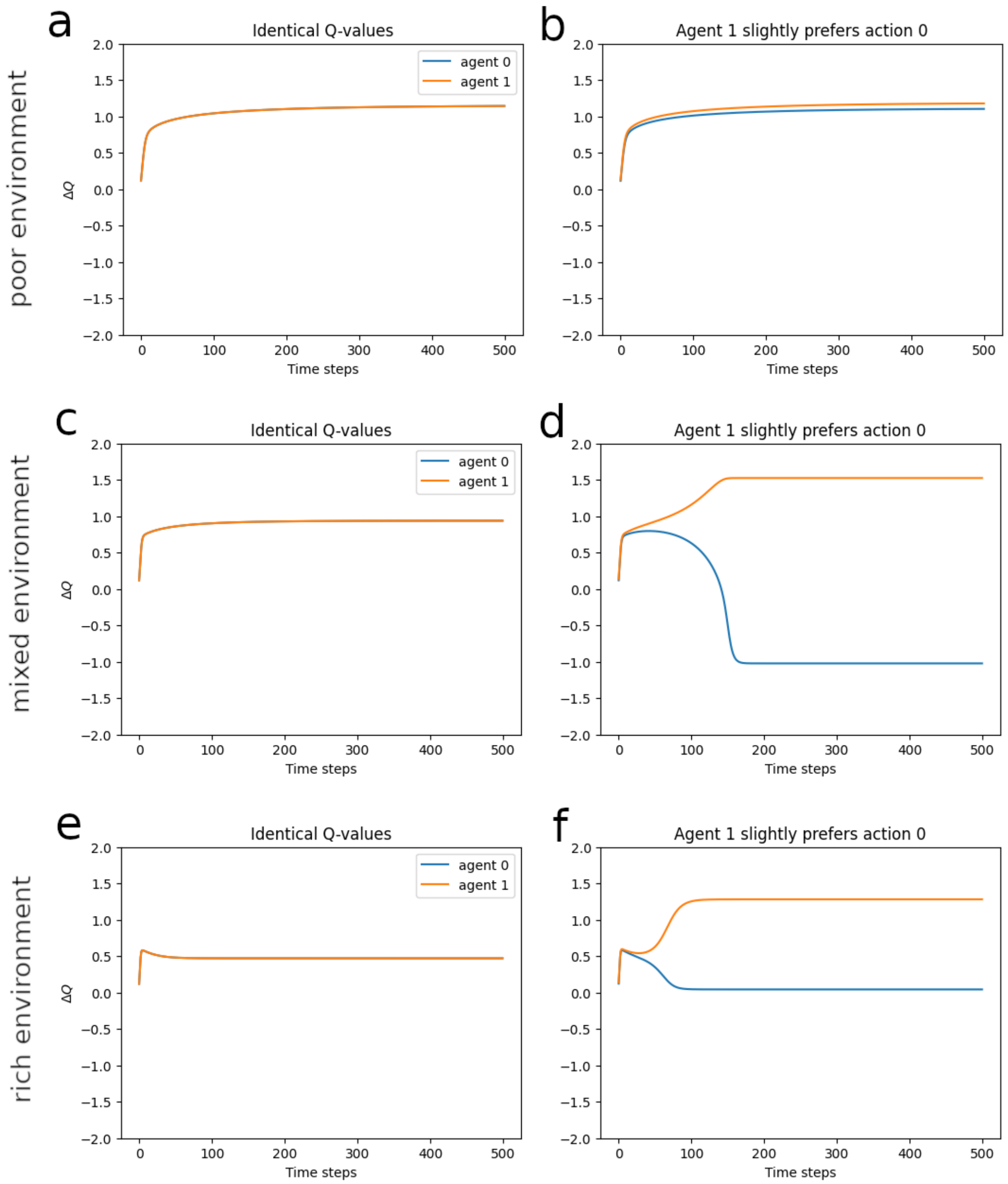

FIG. S5. Agents' Q-value gaps over time according to the deterministic model, when both agents start with the same Q-value gaps (left), vs. when agent 1 starts with a slight preference for the most rewarding action — i.e., $\Delta Q_1 = 0.01$ vs. $\Delta Q_0 = 0$ for agent 0 (right). A-B: in a poor environment; C-D: in a mixed environment; E-F: in a rich environment. The deterministic model was run over 500 trials.
