# Supplementary material for: Moderate confirmation bias enhances decision-making in groups of reinforcement-learning agents
Source: PLoS Comput Biol. 2024 Sep 4;20(9):e1012404. doi: 10.1371/journal.pcbi.1012404 (PMC11404843; doi:10.1371/journal.pcbi.1012404)
Supplement: S6 Fig — (PDF) [file pcbi.1012404.s007.pdf]

S6 Fig. Final polarization as a function of inverse temperature.

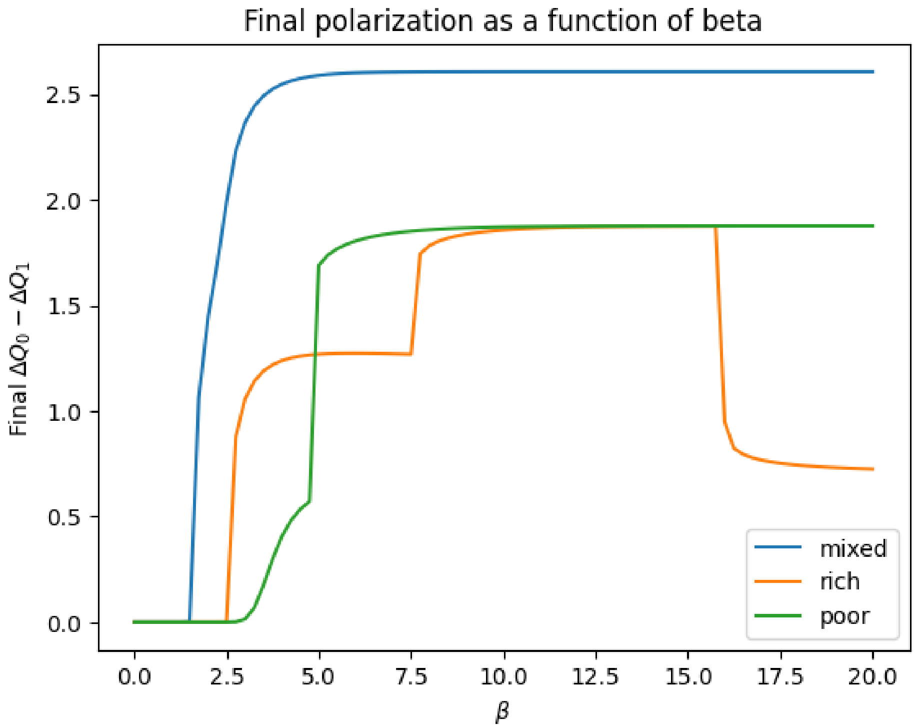

FIG. S6. Final polarization (measured as the difference between agent 1's and agent 2's Q-value gaps) as a function of inverse temperature, in the three environments. Q-value gaps were obtained using the deterministic model defined in Section IV A 4.
