# Supplementary material for: Moderate confirmation bias enhances decision-making in groups of reinforcement-learning agents
Source: PLoS Comput Biol. 2024 Sep 4;20(9):e1012404. doi: 10.1371/journal.pcbi.1012404 (PMC11404843; doi:10.1371/journal.pcbi.1012404)
Supplement: S7 Fig — (PDF) [file pcbi.1012404.s008.pdf]

S7 Fig. Performance in all environments using different inverse temperatures β.

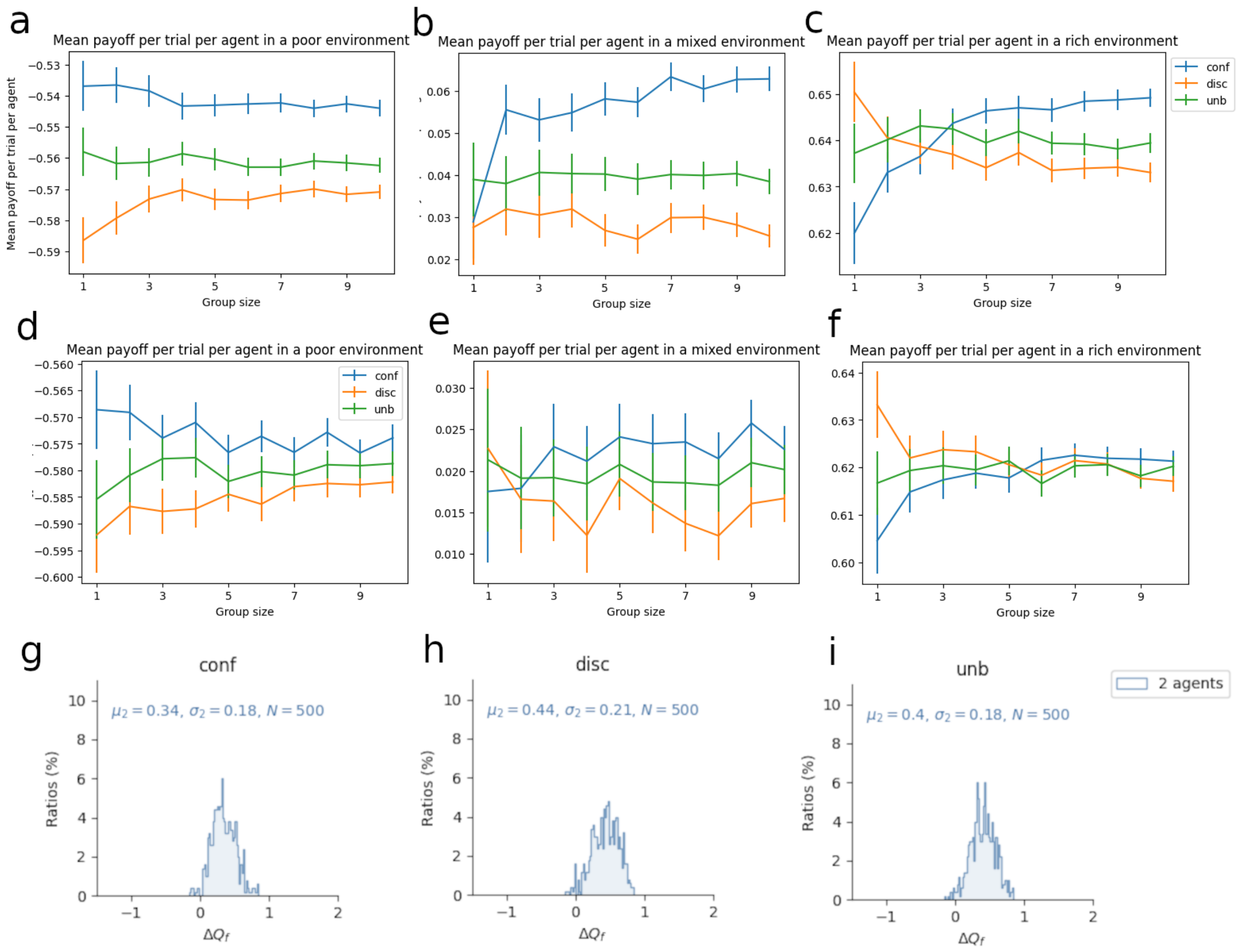

FIG. S7. Performance in all environments using different inverse temperatures β. A-C: mean performance in the three environments with $\beta = 1$. D-F: mean performance in the three environments with $\beta = 0.5$. G-I: final Q-value-gap distributions in a rich environment for the three bias types ($b_{conf} = 3$, $b_{disc} = \frac{1}{3}$, $b_{unb} = 1$) with $n = 2$ and $\beta = 1$.
