# Supplementary material for: Moderate confirmation bias enhances decision-making in groups of reinforcement-learning agents
Source: PLoS Comput Biol. 2024 Sep 4;20(9):e1012404. doi: 10.1371/journal.pcbi.1012404 (PMC11404843; doi:10.1371/journal.pcbi.1012404)
Supplement: S8 Fig — (PDF) [file pcbi.1012404.s009.pdf]

S8 Fig. Difference between confirmatory and unbiased agents' final Q-value gaps in various conditions.

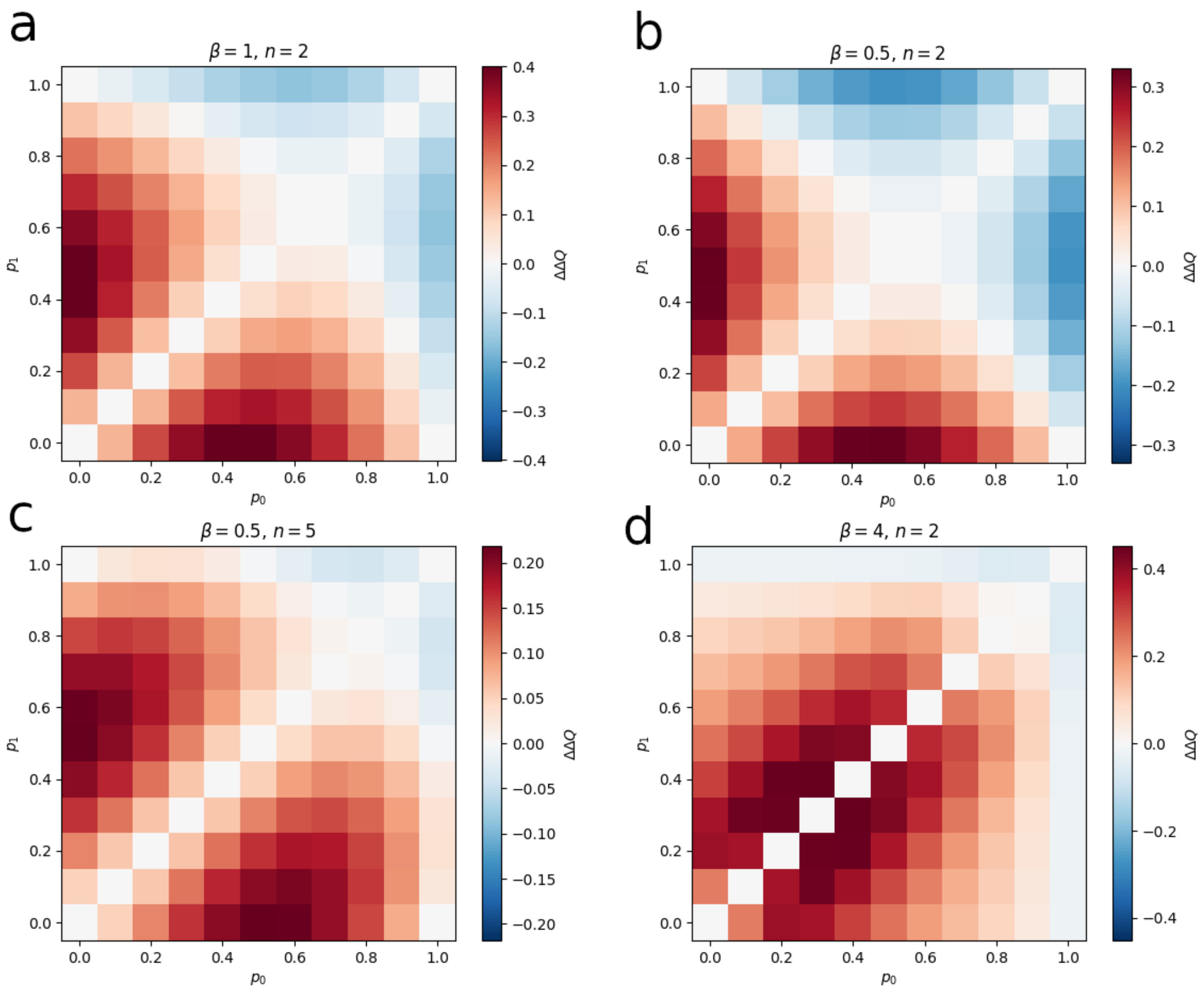

FIG. S8. Difference between confirmatory and unbiased agents' final Q-value gaps (computed with the deterministic model defined in Section IV A 4) in various conditions. A: $n = 2$, $\beta = 1$; B: $n = 2$, $\beta = 0.5$; C: $n = 5$, $\beta = 0.5$; D: $n = 2$, $\beta = 4$. Red denotes higher final Q-value gap (and hence higher performance) in confirmatory agents, blue denotes higher final Q-value gap in unbiased agents.
