# Supplementary material for: Moderate confirmation bias enhances decision-making in groups of reinforcement-learning agents
Source: PLoS Comput Biol. 2024 Sep 4;20(9):e1012404. doi: 10.1371/journal.pcbi.1012404 (PMC11404843; doi:10.1371/journal.pcbi.1012404)
Supplement: S9 Fig — (PDF) [file pcbi.1012404.s010.pdf]

S9 Fig. Evolution of Q-values over time in rich and poor environments, with $\beta = 1$.

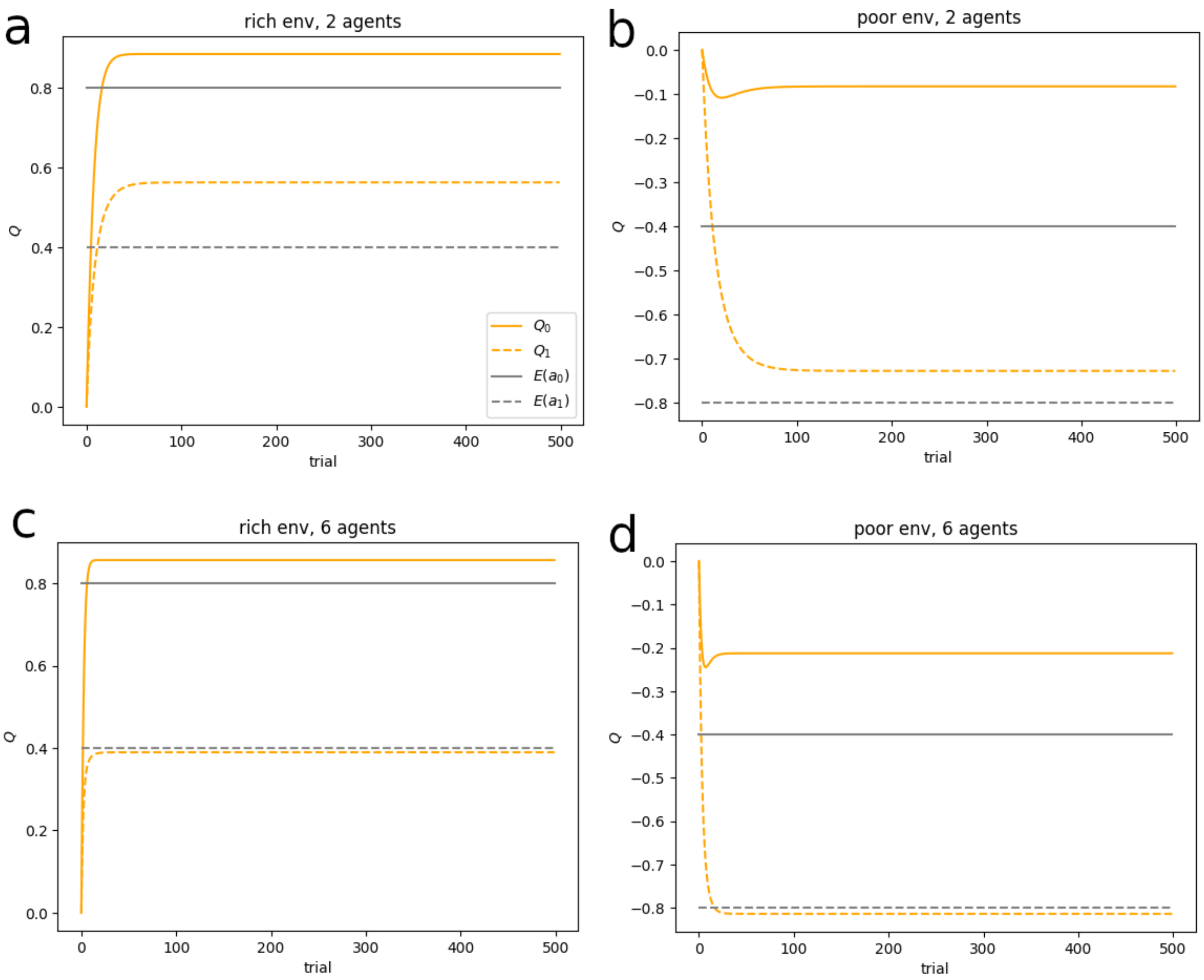

FIG. S9. Evolution of Q-values over time (computed with the deterministic model defined in Section IV A 4) in rich and poor environments, with $\beta = 1$. Gray lines denote the expected values of options 0 and 1, respectively. A-B: $n = 2$; C-D: $n = 5$.
