# Supplementary material for: Moderate confirmation bias enhances decision-making in groups of reinforcement-learning agents
Source: PLoS Comput Biol. 2024 Sep 4;20(9):e1012404. doi: 10.1371/journal.pcbi.1012404 (PMC11404843; doi:10.1371/journal.pcbi.1012404)
Supplement: Attachment — Submitted filename: response_to_reviewers.pdf [file pcbi.1012404.s011.pdf]

Dear Editors,

Hereby we resubmit our manuscript following a substantial revision based on the comments made by the referees. We thank the referees for their thoughtful comments, which helped us improve our manuscript. Below we provide a detailed response to all the comments, and in addition to the revised manuscript file, we also provide a PDF with all changes marked.

With best regards,

Clémence Bergerot, Wolfram Barfuss, Pawel Romanczuk

Reviewers' comments with detailed responses

Reviewer #1: Review of Moderate confirmation bias enhances collective decision-making in reinforcement-learning agents by Bergerot et al.

Major Comments

#1 "collective decision-making"

The authors discuss 'collective decision-making' from the title of the study. However, it appears that no collective decisions are actually made in the various simulations performed. Agents observe the actions of other group members and their associated outcomes, update their individual expectations, and make individual decisions. These individual performances are then aggregated and analysed. Could the authors explain the rationale behind using 'collective decision-making' to describe the decisions made in the simulations? From my understanding, the simulations presented in the paper lead to individual decisions that occur within a social environment and involve social learning.

We agree with the reviewer that no "collective" decisions in a strict sense are made in our setup. The decisions are individual but made in a social context. Therefore, we changed our title to "*Moderate confirmation bias enhances decision-making in groups of reinforcement-learning agents.*" Moreover, we modified all references to "collective decision-making" so that the reader understands we are studying instances of individual decision-making in a social context (or "under social influence").

#2 Mechanistic / computational explanation about the difference in resource scarcity.

The authors found that confirmation bias can be problematic in rich environments, especially in small groups. However, this result remains purely descriptive, and no mechanistic or computational explanation is provided for the origin of this effect. Could the authors provide more information about why this is the case?

We agree with the reviewer that no clear mechanistic or computational explanation has been provided before. Therefore, we explored about this effect in more detail, and found an explanation for the difference in performance between resource scarcities while answering Reviewer 2's comment (1-1).

First, in our simulations, we observed that a larger variance in Q-value-gap distribution – such as the one caused by polarization – resulted in a lower mean in policy distribution and, thus, in performance. The reason for this is the softmax formula's nonlinearity.

However, we also observe polarization, but no drop in performance, in mixed environments. And, when inverse temperature is lower and polarization disappears, small groups of confirmatory agents still exhibit lower performance than unbiased agents (see Fig. R2c, f). This means that polarization is neither necessary, nor sufficient, to explain this lower performance.

What happens in rich environments is that most choice outcomes are (positive) rewards. Therefore, the opportunities to obtain a negative prediction error for the worst option are limited. Moreover, in a small group of agents, the probability that an agent's peer chooses the alternative option is low as well. So, all in all, in a rich environment, the probability that I choose the best option *and* my neighbor chooses the worst option *and* it gets a (negative) punishment is strongly reduced. This lack of negative counterfactual feedback for the worst option results in scarce opportunities to "deflate" the worst option's Q-value – that is, agents end up overestimating both options' Q-values. In a previous study, Cazé and van der Meer (2013) demonstrate that such optimistic updating is not beneficial in rich environments, as Q-value inflation saturates when approaching 1. This results in reduced Q-value gap and, thus, in lower performance.

Therefore, the drop in performance in small groups of confirmatory agents in rich environments is due to two different effects:

- in a consensus regime (low beta): agents do not have enough opportunities to get negative counterfactual feedback and, thus, inflate the worst option's Q-value. They behave optimistically, which is detrimental in rich environments.
- in a polarization regime (high beta): on top of the "optimistic behavior", agents get polarized, with some of them performing very well and some others, very poorly. Because of the softmax formula's nonlinearity, this decreases performance further.

We include the discussion of these mechanisms in a new paragraph in the results section (lines 428-446).

#3 Explanation of why group size counteracts the effect of excessive confirmation bias

The authors also found that group size counteracts the negative effects of strong confirmation bias. However, no computational explanation is provided for why this is the case. Updating with social information appears equivalent to updating from multiple samplings of available options, with the number of samplings equaling the group size. The only difference being that the option preferred by the group would be more sampled. Consequently, it would not be surprising that an increase in group size enhances performance and reduces the negative impact of strong confirmation bias.

With a larger group size, the (weighted) average outcome used to update individual expectations may become increasingly representative of the actual expected value of each

bandit, limiting the likelihood that agents will select and adhere to the worst option. It would be interesting to analyze more closely the relationship between group size and the average outcome received, and how this relationship influences the positive effect of group size on mitigating strong confirmation bias. For instance, it would be beneficial to see a comparison of the average outcomes across groups of varying sizes, which presumably involves a decrease in the noise produced by binary outcomes. I believe this analysis is crucial for understanding the computations in the CARL model and the aforementioned group size effect.

This comment is related to the previous one. When group size increases, counterfactual negative feedback becomes more likely, even in a rich environment. This is why, on Fig. R4c–d, one can observe that agents do not inflate the worst option's Q-value anymore, but slightly deflate it.

To address these very important comments, we discussed these effects extensively in an Appendix in the Supplementary information (lines 838-917). We also added a summary of these effects in the Results section (lines 428-446).

#4 Unstable environment.

In the study, the authors manipulate the environment in one way, specifically through the scarcity of resources. Another common manipulation in a bandit setting is the (in)stability of the environment, where probabilities may reverse during the run. Surprisingly, environment stability remains unexplored in this study. On one hand, a biased agent would typically struggle to switch its decisions toward a newly optimal option in a changing environment. On the other hand, a group of agents biased towards both options (as is the case in some simulations presented here, where performance decreases) may potentially cope better with probability reversal. The diversity in strategies within these groups, which impedes performance in a stable environment, may counterintuitively enhance it in an unstable one.

We agree with the reviewer that the question of instability is important to address, and share their intuition that confirmatory agents would adapt more slowly to change.

Note that, in their simulation study about individual agents, Lefebvre *et al.* (2022) showed that a confirmation bias was beneficial even in bandits with reversals and in bandits where the arms' reward probabilities followed a random walk. However, several related questions still need to be addressed, such as the impact of reversal rate (slow vs. fast fluctuations), the impact of bias heterogeneity (e.g., groups with a mix of biased and unbiased agents) on adaptation to change, etc.

While we believe our model constitutes a good baseline to explore these questions, they open a whole new set of broad avenues for future research. In our opinion, the different aspects of dynamic environments should be investigated systematically and thoroughly, which goes beyond the scope of this work. Therefore, already in the previously submitted version of the manuscript, we outlined the need for further research on these topics in our Discussion, under "Limitations of the present study", lines 560-565.

#5 Agency and confirmation bias in the CARL model

As mentioned in the manuscript, Chambon et al. found that agency is essential for activation

of confirmation bias, and that observational learning alone does not appear to involve any confirmation bias in humans. In the current simulations, most of the information used by agents (depending on group size) is acquired through observation. One might then question whether the assumption made in the CARL model, that the level of bias is similar for one's own outcomes versus those of other agents, is supported by the literature. I believe this is an important aspect of the study and should be addressed in the discussion.

The reviewer is right to mention that, in Chambon *et al.*'s paper from 2020, agency is crucial: participants exhibit a confirmation bias in free-choice trials only. However, in a complete-feedback scenario, participants get feedback about the option they have chosen (factual feedback), and feedback about the option they have not chosen (counterfactual feedback). In free-choice trials, agents apply a confirmation-biased pattern of learning rates on both factual and counterfactual pieces of information.

One of the starting points for our study was that, in nature, we rarely get such counterfactual feedback on its own. To obtain information about alternative options, we must either explore them at a later time point, or observe our peers as they choose these other options. Thus, this latter form of social learning can be seen as offloading the task of obtaining counterfactual feedback on one's peers. In our simulations, there are no forced-choice trials: at each trial, agents choose an option, and get counterfactual feedback by observing other agents' actions and rewards. In this sense, our simulations resemble very much Chambon *et al.*'s free-choice scenario. This is why, from the start, we found that it made sense to apply the same kind of confirmation bias as Chambon *et al.* do.

Moreover, in Chambon *et al.*'s forced-choice trials, participants observe choices that are made by a computer. Research has shown that, in a wide range of interactions, humans seem to behave differently with machines than they do with other humans; in particular, their social response tends to be diminished when interacting with machines, vs. when interacting with human peers [1]. Therefore, we believe it cannot be concluded, from Chambon *et al.*'s study, that observational learning (in the sense of social learning, i.e. learning from other humans) does not involve any confirmation bias. To test this hypothesis, one would need to conduct similar bandit experiments involving social learning, with two or more human participants performing the task at the same time. To our knowledge, such experiments have not been carried out so far.

Although we do not think Chambon *et al.*'s findings contradict our model, we believe the reviewer's comment is relevant with respect to purely observational scenarios – that is, when an agent gathers information from its peers' choices, while refraining itself from making a decision. Indeed, it is not obvious whether a confirmation bias would apply in this case. We added a short section about this question in the Discussion, lines 568-576.

[1] Chugunova, M., & Sele, D. (2022). We and It: An interdisciplinary review of the experimental evidence on how humans interact with machines. *Journal of Behavioral and Experimental Economics*, *99*, 101897.

Minor Comments

In the first Results section, the authors refer to the CARL model details in the Methods section. However, it would help the reader to gain insights about the update process directly in the Results section. Similarly, the softmax temperature is mentioned only in the Methods section. Considering its close connection to the values of learning rates, it would be beneficial for the reader to know its value for each simulation in the Results section.

We indeed did not give much insight about the update process in the Results section. To remedy this issue, we added a paragraph (lines 149-160) at the beginning of the section which, we hope, introduces the model better. Moreover, we added a few lines about the inverse temperature and its value in all simulations (lines 175-179).

Reviewer #2: Dear Editor,

Major points:

(1-1) Test a wider range of beta, and connect it to the previous literature

The results reported seem to rely fully on inverse temperature (beta) being 4, which I believe is very high. In other words, agents assumed here were basically very exploitative (i.e. greedy). I guess their results would not change qualitatively even if they assumed a perfectly greedy choice policy (that is, beta = ∞). However, my huch is that their results would change dramatically under smaller beta values. The interpretation of the results seemed to depend heavily on this assumption.

The reason behind this prediction is that what they found here seems to be very similar to a phenomenon called the "hot stove effect" (Denrell 2007, March 1996). Although the classic hot stove effect assumed a symmetric (unbiased) learning rates, the mechanism is similar to the current study, that is, the long-lasting underestimation due to limited exploration. As I descried in the summary paragraph above, the group size effect came from the fact that agents in a group were able to access information from unchosen options, while solo individuals need to revisit the unfavourable option in order to learn it. Naturally, such limited information sampling is more pronounced when the choice policy is exploitative.

If the results were indeed explained well by such a biased sampling, the found effect of environmental richness, group size, and confirmation bias could be seen as one of possible ways to tweak the dynamics of information gathering. Then, the current work would become well connected to some of the previous literature including Denrell & Le Mens (2007; 2017); Toyokawa & Gaissmaier (2022).

It would be great if the authors can run additional analyses for other range of beta values and can provide an unified view, hopefully explaining a range of phenomena found in the current study as well as the previous finding.

Denrell J (2007) Adaptive learning and risk taking Psychological Review 114:177–187.

Denrell JLe Mens G (2007) Interdependent sampling and social influence Psychological Review 114:398–422.
Denrell JLe Mens G (2017) Information Sampling, Belief Synchronization, and Collective Illusions Management Science 63:528–547.
March JG (1996) Learning to be risk averse Psychological Review 103:309–319.
Toyokawa W, Gaissmaier W (2022) Conformist social learning leads to self-organised prevention against adverse bias in risky decision making eLife 11:e75308.

We appreciate the reviewer's interest in our work, as well as their detailed explanation for the various effects found in our study. Moreover, we are grateful for the literature they provided about the extremely interesting "hot stove effect".

First of all, we would like to clarify that the relatively high inverse temperature of beta=4 was chosen after consulting previous experimental studies with a similar bandit setup. In Lefebvre *et al.* 2017 (studying optimism in individual agents), Palminteri *et al.* 2017 (studying confirmation bias in individual participants), and Chambon *et al.* 2020 (studying choice-confirmation bias), reported fitted betas vary from 2.7 to 7.7, with a mean of approximately 4.1. However, we recognize that, without any clarification, this choice can seem arbitrary, which is why we added this justification in the article's text (lines 177-179).

That said, we agree with the reviewer that the bifurcation in belief formation seems to stem from a lack of exploration, which, in larger groups, is compensated by other agents' choices. Therefore, we first used our deterministic model to get an overview of how beta, as well as the initial difference in Q-value gaps between agents, impact this difference after convergence in a group of two agents. Note that, the higher this difference, the higher the polarization. We varied beta from 0 to 20, and found an interesting landscape of transitions between polarization regimes in all environments. As can be seen in Fig. R1, agents exhibit consensus for low betas in all environments, but end up getting polarized in all environments after a critical beta. For beta=4, which we explored in our study, polarization is much lower in poor environments than in mixed and rich ones. But, after beta=5, agents in a poor environment become significantly more polarized.

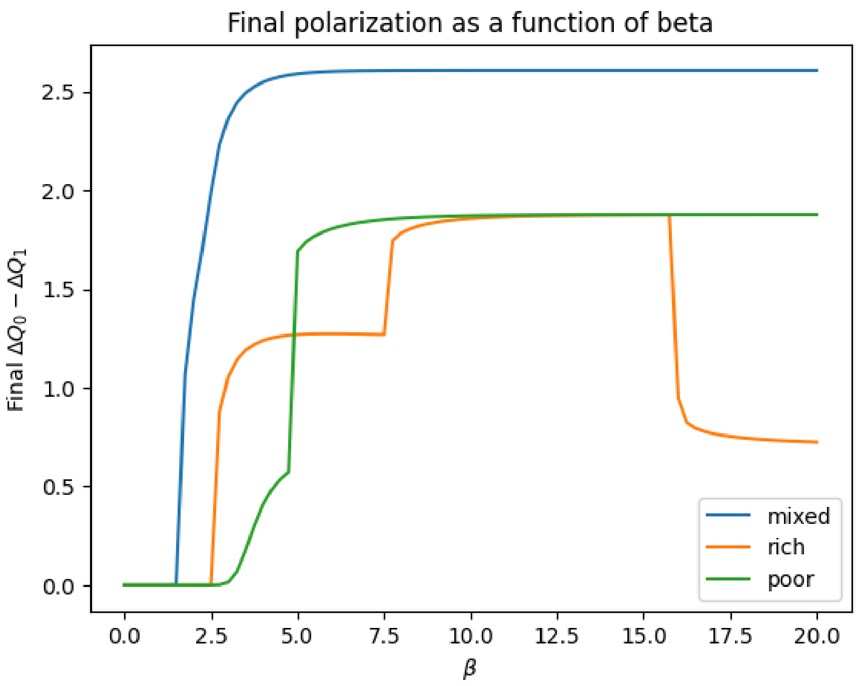

Fig. R1: Final polarization (measured as the difference between agent 1's and agent 2's Q-value gaps) as a function of inverse temperature, in the three environments. Q-value gaps were obtained using the deterministic model.

From there, it is natural to ask how the agents perform when beta is sufficiently low to prevent polarization through increased exploration. Thus, we ran additional simulations with lower betas, in the same conditions as in the article's Fig. 1 (although we varied group size from 1 to 10 instead of 1 to 20, as it did not seem necessary to go further). The inverse temperatures we investigated were beta=1.0 and beta=0.5. As seen in Fig. R1, these inverse temperatures should not give rise to polarization in any environment. We predicted that, because these lower betas reintroduce exploration into the system, agents would more easily get unstuck from suboptimal performance regimes, which, in turn, would cause small groups of confirmatory agents to perform significantly better in rich environments. Results are shown in Fig. R2.

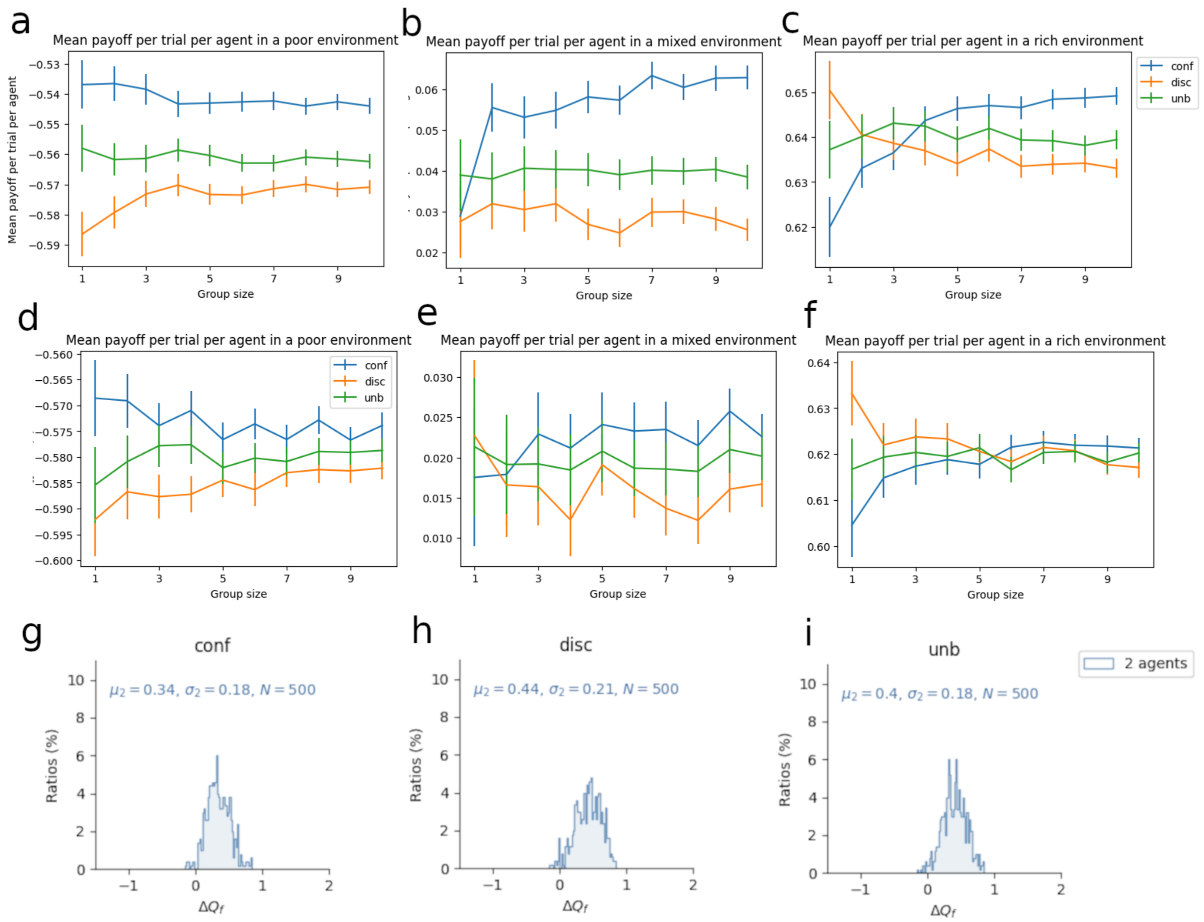

Fig. R2: a-c: performance in all conditions for beta=1.0. d-f: performance in all conditions for beta=0.5. g-i: final Q-value distributions in a rich environment when n_agents = 2 and beta = 1.0.

Analysis of final Q-value gap distributions suggests that, for these lower betas, polarization indeed disappears in all environments. However, as shown in Fig. R2c, small groups of agents with a confirmation bias still appear to underperform in rich environments, although this effect becomes barely visible for beta=0.5 (Fig. R2f).

A more thorough analysis with the deterministic model confirms that lower inverse temperature does not advantage small groups of agents in rich environments (see Fig. R3). In fact, despite the absence of polarization, confirmatory agents in rich environments end up with a lower Q-value gap than the difference between both options' expected values. Observing agents' final Q-values in a poor, and then in a rich, environment (see Fig. R4), one notices the following:

- In the rich environment, the Q-value of the best option is slightly inflated compared to its expected value; whereas the Q-value of the worst option is very much inflated.
- In the poor environment, the Q-value of the best option is very much inflated compared to its expected value; whereas the Q-value of the worst option is only slightly inflated.

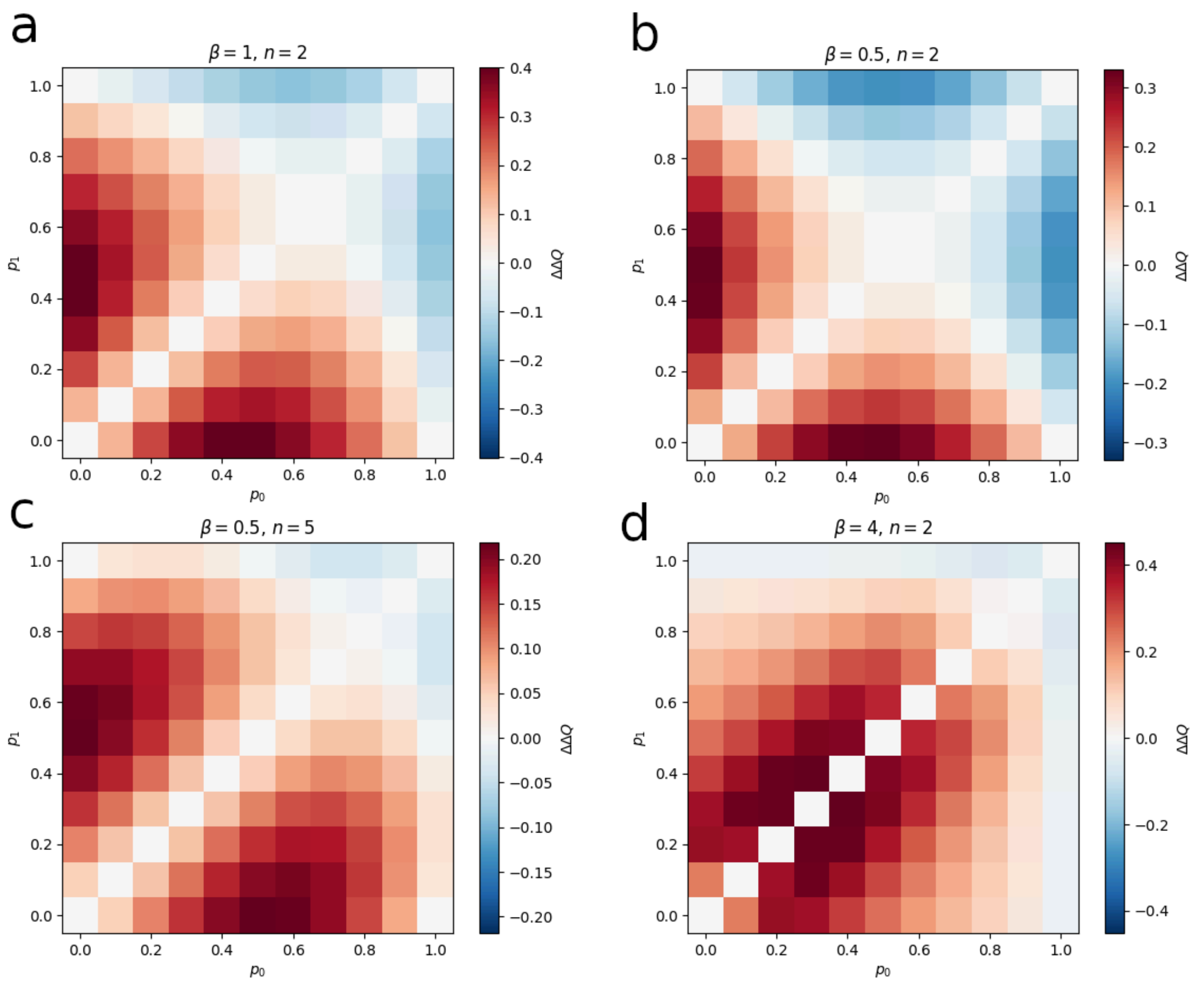

Fig. R3: Comparison between confirmatory and unbiased agents' final Q-value gaps (with deterministic model) in various conditions. a. 2 agents, beta = 1; b. 2 agents, beta = 0.5; c. 5 agents, beta = 0.5; d. 2 agents, beta = 4. Red indicates higher final Q-value gap in confirmatory agents, blue denotes higher final Q-value gap in unbiased agents.

In their simulation study, Lefebvre *et al.* (2022) show that, for individual agents, a confirmation bias is advantageous because it allows an individual to overestimate the best option's Q-value, while underestimating the worst option's Q-value. Here, the aforementioned results suggest that this is not the case for our agents: by inflating both options' Q-values, they behave more like optimistic agents. This is reminiscent of another simulation study, by Cazé and Van der Meer (2013). In this article, the authors study the impact of optimism and pessimism on single agents' performance in a two-armed bandit task. They demonstrate that optimism is advantageous in poor environments, while pessimism is advantageous in rich environments. The reason for such an effect is mathematical: since Q-values are bounded between -1 and 1, an optimistic estimate of the expected value saturates as it approaches 1, and a pessimistic estimate saturates as it approaches -1. As a consequence, optimistic agents end up with a smaller Q-value gap in the rich domain, but a wider gap in the poor domain; whereas pessimistic agents exhibit the opposite pattern.

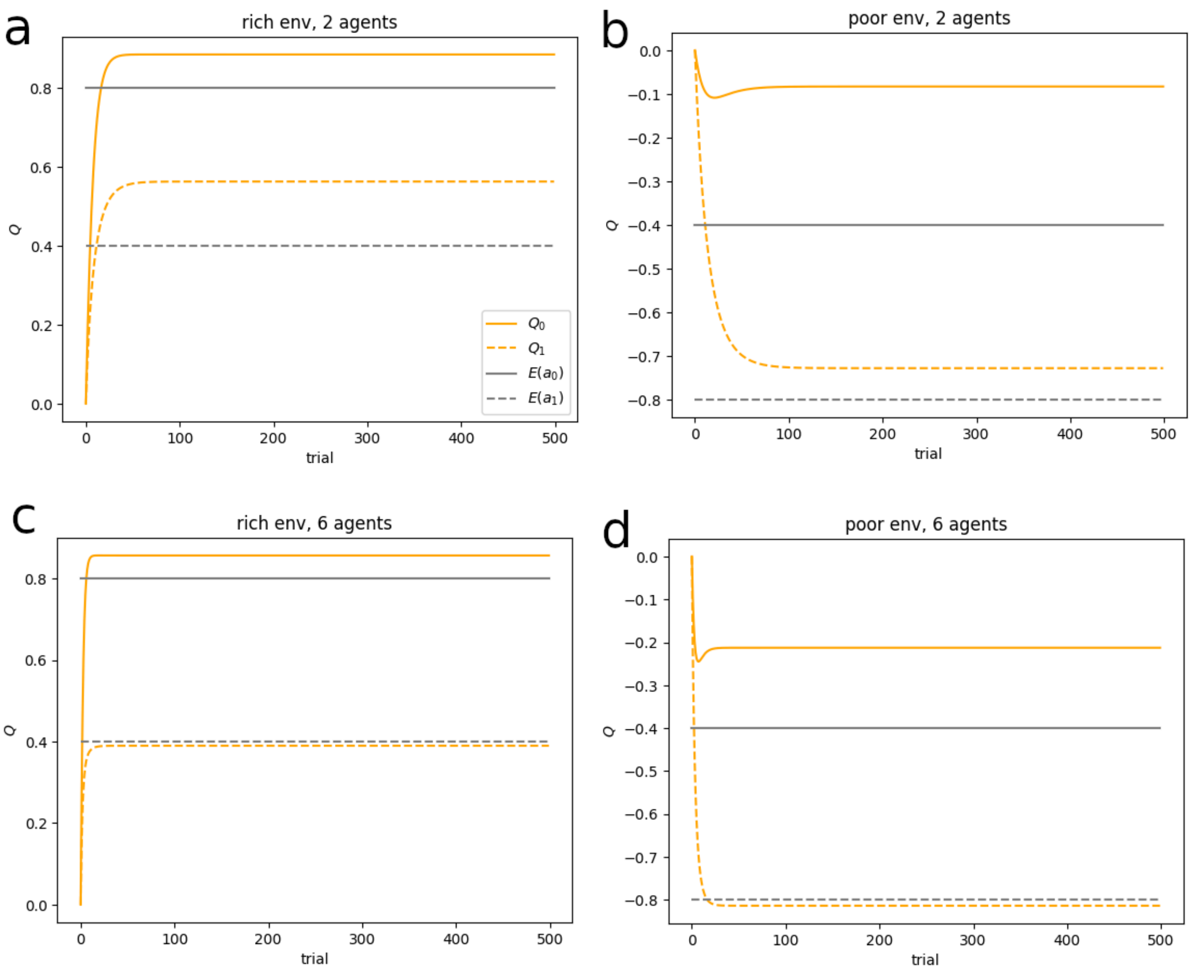

Fig. R4: Evolution of Q-values over time (deterministic model) in rich and poor environments, beta = 1.0. Gray lines denote the expected values of options 0 and 1, respectively. a–b: 2 agents; c–d: 6 agents.

Why does our group of two confirmatory agents behave like an optimistic agent? The reason is that, when it comes to confirmation bias, exploration is not enough: *who* explores matters. Indeed, a confirmatory agent tends to inflate the Q-value of the option it chooses, and deflate the Q-value of the option it does not choose – in short, a confirmatory agent is optimistic about chosen options, and pessimistic about unchosen options. When inverse temperature decreases, an agent tends to sample alternative options more. Subsequently, it has more opportunities to be optimistic about *both* options, thus inflating both Q-values. On the other hand, the opportunities to deflate the other option's Q-value are rare: this happens when the second agent chooses the latter option and receives an outcome of -1, which, in a rich environment, does not happen often. Therefore, the lower the inverse temperature, the more an agent becomes optimistic about the worst option; this results in a narrower final Q-value gap.

When one increases group size (see Fig. R4), one observes that the lowest Q-value is not inflated anymore. Indeed, with a sufficiently large group in a rich environment, an agent has more opportunities to deflate the worst option's Q-value – it is more likely that any other agent will choose that option and get an outcome of -1.

To sum up, the drop in performance in small groups of confirmatory agents in rich environments is due to two different effects:

- in a consensus regime (low beta): agents do not have enough opportunities to get negative counterfactual feedback and, thus, inflate the worst option's Q-value. They behave optimistically, which is detrimental in rich environments.
- in a polarization regime (high beta): on top of their "optimistic behavior", agents get polarized, with some of them performing very well and some others, very poorly. Because of the softmax formula's nonlinearity, this decreases performance further.

Finally, one may wonder how these results are related to the interesting "hot stove effect" the reviewer mentioned. As the reviewer stated and as is explained in Denrell & March, 2001 [2], the hot stove effect occurs when, because of negative outcomes during the first trials, agents undervalue an option and end up neglecting it, although sampling it would correct this undervaluation. As we have shown before, the drop in performance for small groups of agents in a rich environment does not seem to stem from reduced sampling *per se*, but from reduced *counterfactual* sampling. This reduced counterfactual sampling results in optimistic updating of both Q-values, which shrinks Q-value gaps in highly rewarding environments.

To discuss all these effects, we:

- wrote an Appendix containing the previous figures and explanations (lines 838-917 for the text, p. 42-45 for the figures);
- added a paragraph in the Results section (lines 428-446);
- added a paragraph (with citations to the literature the reviewer mentioned) in the Discussion section (lines 523-534).

(1-2) What about other biases? How sensitive the current results are to the model's form?

Perhaps the author can think this is rather a future direction, but I wondered how their results might change by modifying the model's assumption. For instance, as mentioned in the Introduction, there are many different ways to implement asymmetry in learning rates, such as positivity bias (where alphas are implemented separately between positive and negative prediction error) or egocentric bias (alpha for self reward and alpha for social observation). Also, asymmetric alphas could act interactively with a choice inertia (Sugawara & Katahira, 2021), which could impact on the pattern found here. In particular, a positivity bias (and I think the choice-confirmation bias too) can also generate a choice inertia, and Sugawara & Katahira (2021) suggested that assuming a "choice trace" in softmax decision making would allow us to distinguish those two different mechanisms of choice inertia.

I think it would be fine for now to say such modifications could be put into the future direction, but it would be much appreciated if the authors can run some robustness tests using some minor tweaks in model assumptions. It is important to know which part of the results could be generalisable while other parts might be caused by a unique feature of the current model specification.

Sugawara, M., Katahira, K. Dissociation between asymmetric value updating and perseverance in human reinforcement learning. Sci Rep 11, 3574 (2021). https://doi.org/10.1038/s41598-020-80593-7

We thank the reviewer for this relevant comment. Indeed, Sugawara and Katahira demonstrated very well that previous experimental results attributed to confirmation bias could stem from a perseverance bias instead. We agree that it would be good to know how our results depend on variants of and other types of biases., but we feel this question is beyond the scope of this current paper. It is, however, an excellent direction for future work. We also added a paragraph about this specific issue in our discussion (lines 584-592).

Minor points:

(2-1) Is "confirmation bias" an appropriate labelling?
I wondered if the labelling "confirmation bias" makes sense.

Yes, when beta was large like 4, agents' decision making was mostly greedy, choosing what they believed was the best. In such circumstances, it may make sense to assume that reward from the chosen option was a confirmatory cue supporting that what they chose was a good one. However, in lower beta cases, agents often choose options for exploration (choosing options with lower Q-values). I do not think that reward obtained from an exploratory choice necessarily "confirms" their original belief. In the Introduction, the authors mentioned the "choice-confirmation bias" which seemed to fit much better than just saying the confirmation bias. The authors should use "choice-confirmation bias" entirely in the main text.

We think this is a fair and important point. Initially, we chose the term “confirmation bias” to stay aligned with most of the previous literature, but we believe using “choice-confirmation bias” such as in Chambon *et al.*, 2020, would add to concept clarity. However, it is difficult to do so in the article without introducing cumbersome formulations. Therefore, we stressed in our Results section that we are talking about a “choice-confirmation bias”, but that we refer to it as a “confirmation bias” throughout the article for convenience (lines 161-165).

Reviewer #3:
The following comments are ordered by priority level (from most major to most minor).

- 500 simulations were performed at the beginning, and this number is increased later on (probably to get clean distributions), but the number of trials also changes from the simulations in different environments, to the simulations with different biases, to the anaysis of final Q-value differences. The reasons for such a drastic augmentation in trial number (10-fold from the first to the last simulation !) are unclear.

Indeed, trial numbers vary throughout the study. In Experiment 1 (Fig. 1a–c), there were 100 trials. In Experiment 2 (Fig. 1d–f), there were 200 trials so as to include, in the computation of the mean, more trials from the phase after convergence. In Fig. 1d–f, the means are actually not computed over the last 100 trials, and we now corrected this mistake in the revised version. In Experiments 3 and 4 (Fig. 2, Fig. 3a–f, Fig. 4), there were 1000 trials. The reason is that, in these experiments, we consider final Q-value gaps, and we want to get a

precise estimate of Q-value gaps after convergence. It is possible that 500 trials would have been sufficient to get a good estimate. But, since the parameter scan in these experiments is quite wide, it would have been difficult to determine a convergence point for all the different combinations of conditions. Therefore, we chose 1000 trials in order to be safe.
As for Fig. 3g–i, there were 200 trials, but the results shown stem from the last 100 trials so as to observe the bias' impact after learning has converged; moreover, we measure performance improvement here, and we deem it less crucial to have a precise estimate of such improvement after convergence than a precise estimate of final Q-value gap. As far as performance and performance improvement are concerned, we are just interested in relative quantities, in order to compare different bias types on fixed environments.

In order to clarify these choices, we incorporated the justifications in the figure captions (pages 9, 14, 16).

- Why is the «performance improvement» defined in the methods not used? In the case of 1 agent, which acts as a control of the implementation of the learning algorithms, it seems that the mean payoff barely exceeds the expected performance upon random choice in Figure 1, even after removing the first 100 trials (panels d,e,f). Why is that? Was the learning completed?

The performance improvement measure is used in Fig. 3g–i, in order to better compare the heatmaps across environments.
Regarding Figure 1: Expected performance upon random choice in a poor, a mixed, and a rich environment (resp.) is -0.6, 0.0, and 0.6 (resp.). Given the 95% confidence interval, some learning clearly has occurred in all scenarios where there is just one agent. That said, it is true that, in a rich environment (Fig. 1d–f), one agent with a confirmation bias performs quite poorly (but still higher than upon random choice). The reason is that, according to our model, when there is only one agent, the confirmation bias becomes a positivity bias. Indeed, there is no social information to confirm or contradict the agent's choice, so the agent just updates positive prediction errors with a higher weight than negative prediction errors.
In a previous computational study, Caze and van der Meer (2013) demonstrated that a positivity bias is quite detrimental to a single agent's performance in abundant environments. Therefore, our results are not surprising.

- the computations presented in the methods need more detail. In particular, the proof presented in section 4 of the methods is difficult to understand. It should be at least mentioned that the way that the « deterministic » result is computed is an expectancy (which I am not entirely sure of) to get equation 8.

We agree with the reviewer that the Methods' section 4 can be obscure, especially the path that leads to equation 8. To remedy this issue, we clarified the definition of terms (6) and (7), and specified how we get eq. (8) from them. In particular, we:

- stressed that deriving our "deterministic model" is about computing an expected update, as the reviewer pointed out (lines 687-688, 701-702, 705-708);
- clearly stated that eq. (6) is the expected update of a chosen option, and that eq. (7) is the expected update of an unchosen option (lines 701-702, 704);

- explained that eq. (8) corresponds to a more generic expected update (whether an option is chosen or unchosen), and that we need expressions (6) and (7) to compute it (lines 705-708).

- line 247: why pick n=5 agents for this simulation and not more when the authors state from the previous simulation that the intersection point between strongly confirmatory and unbiased accuracy as a function of group size is between n=4 and n=6 (line 220)? Is it to detect the minimum group size to pass beyond the doubled-peak distribution of final Q-value gaps? If so, it should be mentioned in the text.

The reviewer is right to point out that n=5 corresponds to the intersection point between strongly confirmatory and unbiased accuracy, and that it may seem unjustified not to pick a larger group size if one wants to investigate the reason why larger confirmatory groups outperform unbiased ones in rich environments. Our aim during this experiment was neither to show why larger confirmatory groups outperform unbiased groups, nor, as the reviewer suggests, to detect the minimum group size to pass beyond the double-peak distribution of final Q-value gaps. Rather, we just wanted to study why performance improved as group size increased in a rich environment, and group sizes 2 and 5 seemed sufficiently far apart for the distributions to exhibit differences. We believe that, with n=5, we could already show what contributes to such performance improvement – i.e., the decrease in bimodality, which correlates with a decrease in the distribution's variance, even for strongly confirmatory agents. Nonetheless, we ran additional simulations with a group size of n=8 agents for completeness (see Fig. R5). The resulting distributions are qualitatively similar to our previous distributions for 5 agents but, as expected, they exhibit a higher mean and a lower variance in the biased cases (Fig. R5 b. and c.).

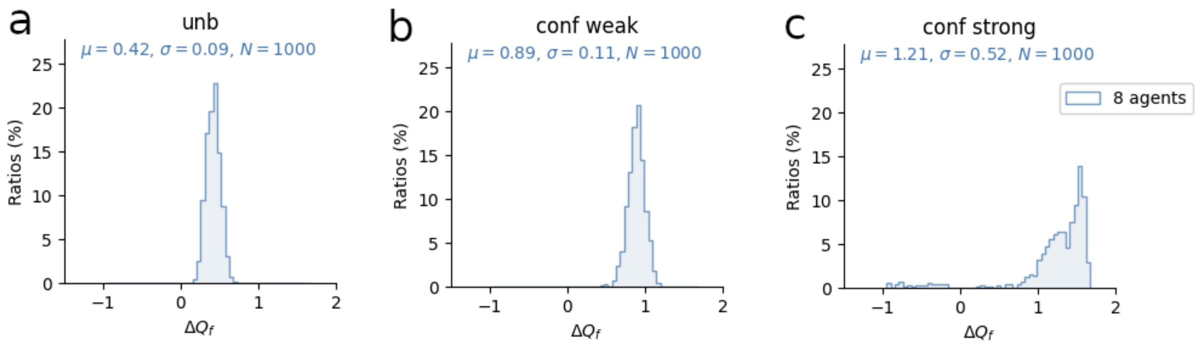

Fig. R5: distributions of final Q-value gaps for one agent in a group of 8 agents.

Since, in our opinion, the relevant effects are already explained with n=5, we decided to keep the latter group size for the simulations in our article. However, we added a few sentences to clarify why we chose n=5 rather than another group size (lines 271-274).

- Why choose to show the mean payoff instead of the average probability of picking the correct answer, which would be closer to the usual representation of a learning curve?

In Fig. 1, 2, and 3g–i, we chose this measure because this is what has been done in previous simulation studies (e.g., Lefebvre *et al.*, 2022). The probability of choosing the best option is shown in Fig. 3d–f. Note that the curves we have represented are not learning

curves: we plot mean performance as a function of group size, or of bias strength. We did not investigate the impact of the bias on the learning phase/learning curve, as we are interested in performance after convergence on a static choice task.
Nonetheless, we plotted the average probability to pick the correct answer in the same conditions as in the paper's Fig. 1 (see Fig. R6). As expected, this average probability correlates with the average payoff. Both are measures of performance.

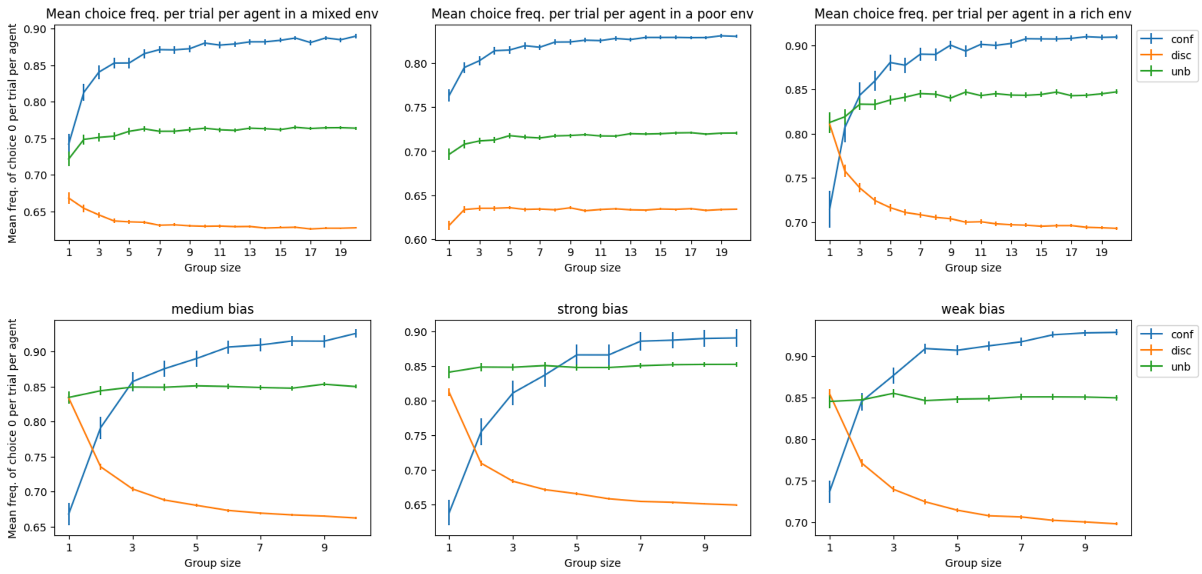

Fig. R6: Average probability of picking the best option, conditions are the same as in the paper's Fig. 1.

Because the correlation is quite clear, and because plotting mean collected rewards is in a continuity with previous literature (e.g., Lefebvre *et al.*, 2022), we decided not to add changes to the manuscript.

- In Figure 1, b is defined in the a,b, and c panels (referenced from section II A) when only introduced in section IIB in the text. I'd recommend defining it early on in section IIA.

We thank the reviewer for drawing our attention to this issue. We introduced b's definition in section IIA (lines 169-170).

- In Equation (5) of the methods, the * (convolution) should be replaced with an x (multiplication: \times in LaTeX and Word) or with a dot, as in the rest of the equations.

We agree, and made the corresponding change (line 677).

- The notations are not always consistent throughout the text: the number of agents is sometimes denoted n (as in section II A) and sometimes N_agents (as in section II C).

We agree; all instances of N_agents were replaced by n.

-Similarly, the policy is sometimes referred to as X^i_a(t) (equation 5) and sometimes as X^i_t(a) (methods section 4). While it is understandable that these two notations are used in

different contexts, they refer, in the end, to the same quantity. Using the same or different notations are two valid takes. Still, considering the journal's broad readership, and this research in particular being of potential interest to scientists from various fields, consistency might bring clarity to the paper.

We agree; we replaced all instances of X^i_t(a) with X^i_a(t).

- At the end of the methods, the reward probabilities associated with each environment could be written again to obtain a self-standing methods section and for better clarity.

We implemented these suggestions (lines 724-729).
